# Dispersal of Amur tiger from spatial distribution and genetics within the eastern Changbai mountain of China

**DOI:** 10.1002/ece3.4832

**Published:** 2019-02-18

**Authors:** Yao Ning, Aleksey V. Kostyria, Jianzhang Ma, Marina I. Chayka, Valentin Yu Guskov, Jinzhe Qi, Irina N. Sheremetyeva, Meng Wang, Guangshun Jiang

**Affiliations:** ^1^ Feline Research Center of Chinese State Forestry Administration, College of Wildlife Resources Northeast Forestry University Harbin China; ^2^ Federal Scientific Center of the East Asia Terrestrial Biodiversity Far Eastern Branch of Russian Academy of Sciences (FSCEATB FEB RAS) Vladivostok Russia; ^3^ WWF‐Russia, Amur Branch Vladivostok Russia

**Keywords:** Amur tiger dispersal, genetic distances, migrant detection, spatial distribution

## Abstract

Population dispersal and migration often indicate an expanded habitat and reduced inbreeding probability, and to some extend reflects improvement in the condition of the population. The Amur tiger population in the northern region of the Changbai mountain in China mostly distributes along the Sino–Russian border, next to the population in southwest Primorye in Russia. The successful dispersal westward and transboundary movement are crucial for the persistence of the Amur tiger in this area. This study explored the spatial dispersal of the population, transboundary migration, and the genetic condition of the Amur tiger population within the northern Changbai mountain in China, using occurrence data and fecal samples. Our results from 2003 to 2016 showed that the Amur tiger population in this area was spreading westward at a speed of 12.83 ± 4.41 km every three years. Genetic diversity of the Amur tiger populations in southwest Primorye was slightly different than the population in our study area, and the potential individual migration rate between these two populations was shown to be about 13.04%. Furthermore, the relationships between genetic distances and spatial distances indicated the existence of serious limitations to the dispersal of the Amur tiger in China. This study provided important information about spatial dispersal, transboundary migration, and the genetic diversity of Amur tigers in China, showed the urgent need for Amur tiger habitat restoration, and suggested some important conservation measures, such as corridor construction to eliminate dispersal barriers and joint international conservation to promote trans‐boundary movement.

## 1 INTRODUCTION

Dispersal is the movement of an individual from its birth site or group to its mating site(s) or group(s) (Coulon et al., 2004). It plays an important role in speciation (Ronquist, 1997) and is a major determinant of population dynamics (Cowen & Sponaugle, 2009). Dispersal changes the spacing patterns of animal distribution (Bowman, Jaeger, & Fahrig, 2002) and results in range expansion (Kokko & López‐Sepulcre, 2006). Thus, dispersal is important for resisting range shrinkage and sustaining sink populations (Mouquet & Loreau, 2002; Zhan et al., 2007). Dispersal also influences the level of gene flow and gene frequency (Neubert & Caswell, 2000; Tackenberg, Poschlod, & Bonn, 2003), as it promotes individual exchange between populations and invokes inbreeding avoidance, assuming that reproduction occurs following dispersal (Handley & Perrin, 2007). In general, successful animal dispersal reflects a positive situation for the population and can be used as an indicator for wildlife recovery. Therefore, dispersal should be a major research focus in conservation biology and evolutionary biology (Berry, Tocher, & Sarreb, 2004).

Microscale genetic differentiation is a good tool for quantifying dispersal and revealing spatial patterns in certain conditions (Coulon et al., 2004; Zhan et al., 2007). In attempts to better understand the dispersal of a species, much research focused on the relationship between genetic and geographical distance. For example, Hansen and Mensberg (1998) found a significant correlation between geographical and genetic distances in the anadromous brown trout (*Salmo trutta*). Trizio et al. (2005) found that on a large scale, a significant correlation between the genetic and geographical distance of red squirrels (*Sciurus vulgaris*), suggesting that geographical factors limited the dispersal of this population.

The Amur tiger (*Panthera tigris altaica*) is an flagship species and before the 20th century was widely distributed in northeastern China, the Korean Peninsula, and the southern part of the Russian Far East (Sugimoto, Nagata, Aramilev, & McCullough, 2012; Dou *et al.,* 2016). After the middle of the 20th century, excessive poaching, habitat loss, and habitat fragmentation led to a rapid decrease in population numbers and habitat range (Wang, Feng, et al., ; Sugimoto et al., 2012; Dou *et al., *2016). Now, fewer than 400 Amur tigers survive in Russia and the eastern region of northeastern China (Kerley et al., 2015). This species has been classified as an endangered species by the International Union for the Conservation of Nature (IUCN; Sugimoto et al., 2012; Peng, Liu, Hou, & Xing, 2016) and protected by Chinese government as a first class protected subspecies. Russia banned tiger hunting in 1974 and implemented measures to protect the population, which led to a slow recovery of the tiger (Miquelle et al., 2007). In 1998, the Chinese government initiated the Natural Forest Protection Program, directed at forest biomass recovery, which increased the habitat area of large cats (Jiang et al., 2017). Prior to this, the Amur tiger habitat in China was in poor condition for a long period and transboundary movement of the tiger from China to Russia was impeded. Fortunately, as the Chinese government invested more money and focused on Amur tiger conservation, the habitat and population began recovering. Reports on transboundary movement and the reproduction of tigers in China have become more frequent in recent years. For example, a female was found to have moved across the Sino–Russian boundary into the Wanda mountain in China (Miquelle et al., 2006) and a breeding family consisting of a female Amur tiger with three cubs migrated to the Hunchun Forestry Bureau of the Changbai mountain in China in 2013 (Jiang, 2014). As the Amur tiger population recovered in China, many researchers began focusing on population dynamics but dispersal and habitat expansion were neglected.

The Amur tiger population in Russia is divided into two main subpopulations: 95% in the Sikhote‐Alin population and a small population in southwest Primorye (Henry et al., 2009). In China, there were more than 26 individuals distributed in 3 main Amur tiger habitat patches, called the Laoyeling mountain, Zhangguangcai mountain, and Wandashan mountain (Wang, Feng, et al., ). The Laoyeling and Wandashan patches are next to the Sino–Russian border, while the Zhangguangcai mountain are the only patch away from the border but only a few individuals live there. It was suggested that the southwest Primorye population in Russia holds critical source populations vital to the recovery of tigers in neighboring China (Miquelle et al., 2010). But the two Russia populations are facing the problems like loss of genetic diversity, diseases, and competition caused by dispersal limitations. Thus, promoting transboundary movement of Amur tiger from Russia to China and dispersal from the Laoyeling and Wandashan patches into Zhangguangcai to extend the Amur tiger habitat and promote individual migration between different populations. This would not only in support Amur tiger population recovery in China; it would also benefit this subspecies. In this study, we firstly collected the occurrence information of Amur tigers in Changbai mountain region in China to investigate the dynamics of population distribution. Secondly, we uncovered evidence of potential migration across the Sino–Russia border areas by assessing the origins of genetic information. Finally, we explored the dispersal consistencies between spatial distribution and genetic distance to guide the dispersal and migration management of the Amur tiger in China.

## 2 MATERIAL AND METHODS

### 2.1 Ethics

Blood samples used in this research were taken from a captive‐bred tiger that had died naturally, provided by the Amur Tiger Park, Harbin. All fecal samples were collected noninvasively, and all permissions necessary to collect and analyze the samples were granted by the Endangered Species Scientific Commission of China. Both Chinese and Russian partners completed field sample collections and DNA extractions independently, and DNA samples extracted in the Russian Federation were delivered to China for further analysis, following the regulations of Convention on International Trade in Rare and Endangered Species (CITES). Total joint analysis with Russia was permitted by both Chinese and Russian governments.

### 2.2 Study area

**Figure 1:**
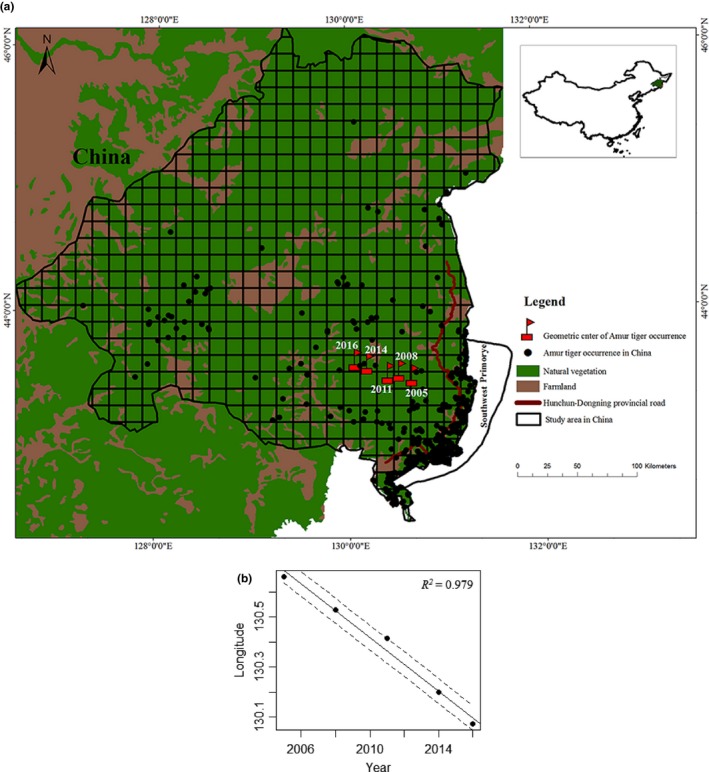
Dispersal dynamics of Amur tiger geometric center points: Black points represent all occurrence the points of the Amur tiger from 2003 to 2016, and red squares represent the geometric centers of the tiger information points between 2003–2005, 2003–2008, 2003–2011, 2003–2014, and 2003–2016 (a), and the positive relationship between the time (year) and longitude of the geometric center of Amur tiger occurrence in China (b)

We restricted our study area to a 93,736.7 km^2^ region in the northern Changbai mountain in China, accounting for 68% of the China tiger distribution area (Figure 1a). The study area covered the main Amur tiger habitat patches of Zhangguangcai mountain and Laoyeling mountain in China, and national nature reserves for the Amur tiger of Hunchun, Wangqing, Huangnihe in Jilin province, and Laoyeling, Muling in Heilongjiang province were included in this area. The Laoyeling mountain patch was connected with the Land of the Leopard National Park in southwest Primorye, Russia. As the Amur tiger population in southwest Primorye in Russia could not dispersal to Sikhote‐Alin in Russia, the Laoyeling mountain patch in China was the first optional area dispersal or migration.

### 2.3 Amur tiger occurrence data

To collect as much Amur tiger occurrence data as possible, we conducted three surveys simultaneously: historical record collection, camera trap survey, and transect survey in winter.

For the historical record collection, we got data from the Feline Research Center of Chinese Forestry Administration (FRC‐CSFA) in China and the local forest bureau. FRC‐CSFA is a specialist agency for Amur tiger and leopard research in China. FRC‐CSFA extracted and recorded all the occurrence information of Amur tigers, such as killings, footprints, and feces as reported by local residents and forestry workers since 2012. Jilin provincial government introduced ecological compensation for ecological conservation in 2001. When livestock was killed by wild animals, the government paid the cattleman for it after professional identification; all these cases were recorded by the local forest bureau. Since 2012, we installed 1,000 camera traps in the study area, all the camera traps were designed at an average density of 1 site/10 km^2^. All Amur tiger occurrence data were extracted with geographical coordinates.

The transect survey in winter was conducted primarily to find traces of the tiger and collect fecal samples. We surveyed different areas each year from 2012 to 2016.

All the occurrence information was divided into groups by every 3 years, given that the data were scarce in some years. Totally, five groups were formed, and the fifth group only included 2015 and 2016. The occurrence locations in each group were located on the map based on geographical coordinates. After that, we divided the study area into grids of 14 km × 14 km. The longitudes and latitudes of center points of the grids where Amur tiger occurred were extracted to calculate the geometrical center of each group. The calculated geometrical centers were used to show the population dispersal of the Amur tiger in China. All analysis was conducted using ArcGIS software (Environmental Systems Resource Institute, ArcGIS 10.3; www.esri.com).

As we want to detect whether the Chinese tiger population was dispersing inland, we also built linear regression models to detect the relationship between time and the average longitudes of the centers for each three‐year interval.

### 2.4 Fecal sample collection and DNA extraction

Fecal samples were collected from 2012 to 2016, mainly by transect survey or following Amur tiger tracks in winter, both in the study area and in the Land of the Leopard in Russia in 2015 (Figure 1a). To maintain the quality of the samples, they were stored in a freezer below −80°C until further analysis. DNA extraction was performed by using the QIAamp DNA stool mini kit following the manufacturer's protocols with minor modifications. For the samples with low quality, the TE buffer solution was decreased to a half volume. We used NanoDrop 2000 to measure the DNA concentration and dilute all the sample concentrations to the working solution of 5 ng/μl. A total of 78 DNA samples were extracted from scat provided by Russia, including 29 species unknown samples and 49 samples suspected as Amur tigers.

Species identification was conducted following the method of Sugimoto et al. (2006). 271 bp cytochrome b sequence was identified in 0.8% agarose gel and visualized on the UV transilluminator. We used KOD FX Neo DNA polymerase (TOYOBO) in a 20 μl system and added to 10 ng DNA with 60°C annealing temperature:

Pta‐CbF (5′‐TTTGGCTCCTTACTAGGGGTG‐3′).

Pta‐CbR (5′‐CCGTAAACAATAGCACAATCCCGATA‐3′).

For individual identification, we used the method described in Zou, Uphyrkina, Fomenko, and Luo (2015) that uses a multiplex STR genotyping system with 22 loci split into 4 panels (Panel A: FCA8, FCA32, FCA69, FCA77, FCA105; Panel B: FCA5, FCA43, FCA90, FCA91P, FCA94, FCA290P; Panel C: FCA44, FCA126, FCA161P, FCA176, FCA220, FCA441; and Panel D: FCA211P, FCA293P, FCA304P, FCA310P, FCA391P; see Supporting information Table S1). We performed three replicates of amplification from 16 of better quality samples. Based on the results, we selected two panels (Panel A and Panel C), which showed less missing data and more defined genotype data (data in black color in Supporting information Table S1). PCR was set up in a 10 μl system with 5 μl of 2× Multiplx PCR Masermix, 1 μl of 10 × primer mix (primer concentration same as Zou *et al.*), and 10–20 ng of DNA by using QIAGEN Multiplex PCR Plus Kit. PCR amplification was performed on a Model 9700 Thermocycler (PerkinElmer) following the process below: 1 cycle for 5 min at 95°C, 35 cycles for 30 s at 95°C, annealing temperature 60°C for 90 s, 72°C for 3 min, and 1 cycle of 68°C for 30 min. The products were analyzed with an ABI 3730xl Automated DNA Sequencer (Applied Biosystems). We performed genotyping four times, after which the genotype was determined by standard peak map. Meanwhile, correspondingly, the consensus genotype was determined, which is found a homozygote with a minimum of three times and two times for a heterozygote (Rozhnov et al., 2013). For the samples that failed to obtain consistent genotype results, we added another three amplifications. We used GeneMapper V4.0 software to analyze the genotypes, and then, Allelogram V2.2 was used to find errors in the genotypes and provides consistent data for the following analysis.

To identify the sex, we amplified the X and Y chromosome fragments in samples that were successfully amplified more than 14 alleles by sex primers ZFX‐PF/ZFX‐PR, DBY7‐PF/DBY7‐PR (Pilgrim, Mckelvey, Riddle, & Schwartz, 2005; Sugimoto et al., 2014). Here, the male tiger DNA isolated from blood was treated as a positive control for distinguishing PCR failure and negative control without DNA for monitoring contamination.

### 2.5 Population genetic analysis

The Excel Microsatellite Toolkit v3.1.1 was used to identify individual tigers. This identification allows all alleles identical or only one mismatch. It was also used to count polymorphism information contents (PIC), as well as observed and expected heterozygosity (*H*_O_ and *H*_E_) (Zhan et al., 2007). The program Micro‐checker (Oosterhout, Hutchinson, Wills, & Shipley, 2004) was used to test the data of null alleles and stuttering or large allele dropout. Multiplex marker potential for correctly identifying individuals was calculated in GIMLETv1.3.3 (Valière, 2002). Deviations from Hardy–Weinberg equilibrium (HWE) and the presence of linkage disequilibrium (LD) between loci were assessed with Genepop 4.2 (Raymond & Rousset, 1995).

### 2.6 Detection of migrants

STRUCTURE v2.3.4 assigned individuals to K clusters based on multilocus genotypes by using the Bayesian approach (Pritchard, Stephens, & Donnelly, 2000). The admixture model with correlated allele frequencies was used, with a burn‐in of 100,000 and 1,000,000 Markov chain Monte Carlo (MCMC) repetitions and 10 iterations per *K* (*K* = 1–10). The method of Evanno, Regnaut, and Goudet (2005) was used to determine the best fit value, as implemented in STRUCTURE HAVESTER (Earl & Vonholdt, 2012; McGlaughlin et al., 2015).

Pritchard et al. (2000) presented a formal Bayesian test to assess whether any individual within the samples was a migrant to this group or a resident. Procedures ran without population information to ensure that the identified population was in line with the genetic information. The USEPOPINFO option of STRUCTURE was employed to estimate the origin of an unknown origin by specifying the source of certain individuals. The number of burn‐ins and total number of replicates were same as above. The option of using population information to test for migration was selected, and the value of MIGRPRIOR in the range was selected from 0.001 to 0.1. Here, only results for MIGPRIOR = 0.05 and set *K* = 2 (from clustering analysis without population information) based on the analysis of STRUCTURE HAVESTER were considered. We identified an individual as admixed by using the criteria of mixed Q and undetermined GeneClass assignment probability and being an F_0 _migrant with a high probability (Bergl & Vigilant, 2007).

In GENECLASS2.0, we assigned or excluded reference populations as possible origins of individuals on the basis of multilocus genotypes (Piry et al., 2015). The assignment threshold was set to 0.05. As recommended, we chose the method of Paetkau, Slade, Burden, and Estoup (2004) as the simulation algorithm and defined the minimum number of simulated individuals as 10,000 (Paetkau et al., 2004).

In GENECLASS, the test of first‐generation migrants was performed using the frequencies‐based and Monte Carlo resampling methods of Paetkau. We used the *L_h_*/*L*_max_ likelihood test statistics for the likelihood computation. An alpha level of 0.05 was determined as a critical value (Li, Lancaster, Cooper, Taylor, & Carthew, 2015; Reddy et al., 2012).

### 2.7 Relationships between genetic and geographical distances

Two methods for individual heterozygosity evaluation were used as follows: (a) homozygosity by loci (HL) and (b) internal relatedness (IR). HL was estimated by weighting the contribution of each locus accounting for differences in the number and frequency of alleles between loci (Aparicio, Ortego, & Cordero, 2006). IR estimated heterozygosity across individuals taking the frequencies of alleles, including rare ones, into account. The HL index varied from 0 (when all loci were heterozygous) to 1 (when all loci were homozygosis). IR varied between 1 and −1. Geographic distances were derived from ArcGIS 10.3. Statistic models were run using R software (available at https://cran.r-project.org).

Many methods can be used to calculate genetic distances, mainly verified by the strength of the relationship between pairwise genetic distances and landscape distances among sampled individuals in a population (Shirk, Landguth, & Cushman, 2017). The Nei's Da distance was thought superior to other genetic distance measures when constructing phylogenetic trees from simulated microsatellite data (Takezaki & Nei, 1996). Therefore, the D_A_ parameters were calculated as genetic distance in this study by using POPULATION (version 3.5). Then, we used ArcGIS 10.3 to measure the Euclidean distance between different individuals in the study area based on feces location and genetic results. In addition, the average distance of each individual to the Sino–Russian boundary was also measured. Finally, we used a model to test the relationship between genetic distance and Euclidean distance.

## 3 RESULTS

A total of 1,083 records of Amur tiger occurrences were collected in this area from 2002 to 2016, including 278 camera trap events, 103 DNA samples (feces), and 702 other records including killings, footprints, ecological compensation, and other traces left by tigers.

We derived 5 geometric center points of occurrence locations for the Amur tiger in China in different periods from 2003 to 2016 (Figure 1a). The geographic distance between the 5 geometric center points indicated that the Amur tiger population was spreading westwards at a speed of 12.83 ± 4.41 km every three years and had moved about 50.15 km totally into inner China from 2003 to 2016. The linear regression model also showed a positive relationship between time (year) and the average longitudes of geometric center points (*R* ^2^ = 0.979, *p < *0.001) (Figure 1b).

Three of the original 11 microsatellite loci (Fca8, Fca126 and Fca441) were discarded because they were monomorphic or there was a low rate of amplification, given that usable combined genotypes must include at least 14 alleles in the whole multilocus genotype. In Russia, nine individuals were identified by the match function of the Microsatellite Toolkit, four individuals were detected once and five were detected 2–4 times. For gender identification by sex‐specific primers, five females and four males were identified. In China, a total of 15 individuals were identified, including nine males, five females, and one sex unknown, and were confirmed after amplification (Table 1). Only one individual was detected both in China and in Russia.

**Table 1:** The total number of fecal samples collected, positive samples, genotyping success, and the number of individuals confirmed in both Russia and China

	Total number of samples collected	Number of positive samples	Number of genotyped	Number of individuals
Russia	78	65	18	9
China	103	93	22	15

**Figure 2:**
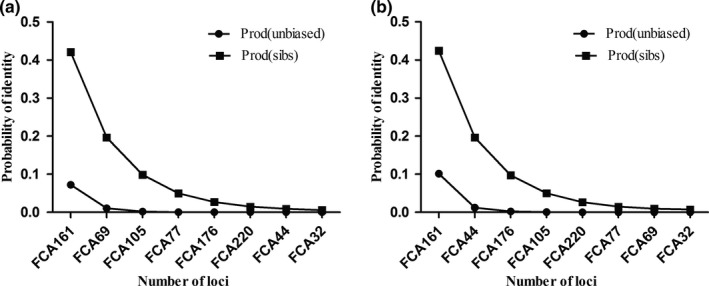
Graphical representation of *P*_ID _(unbiased) and *P*_ID _(sibs) values of eight selected polymorphism microsatellite loci samples from both Russia (a) and China (b)

Expected heterozygosity (*H*_E_ ± *SD*) in Russia (0.61 ± 0.1) was higher than in China (0.57 ± 0.15), while observed heterozygosity (*H*_O_) in Russia ranged from 0.43 (FCA32) to 0.78 (FCA161 and FCA220). In China, it ranged from 0.14 (FCA32) to 0.83 (FCA176). The average polymorphism information content (PIC) per locus was 0.51 in Russia and 0.49 in China. Expected heterozygosity, observed heterozygosity, and polymorphism information for the 8 microsatellite loci are presented in Table 2. The cumulative unbiased *P*_ID_ value of samples from Russia was 6.866 × 10^−7^ (Figure 2a), and for samples in China, it was 4.177 × 10^−6^ (Figure 2b), which accurately identifies individuals within a small population. The micro‐checker analysis detected no null alleles or genotyping. No evidence of scoring error was found due to stuttering, large allele dropout and null alleles at eight loci. This illustrated that the microsatellite genotyping was reliable. When all the samples were pooled together into a single population, both Chinese and Russian samples showed no significant deviations from the Hardy–Weinberg equilibrium and linkage disequilibrium was found at 8 loci after sequential Bonferroni correction.

**Table 2:** Eight polymorphic microsatellite loci screened on 9 tiger individuals from Russia and 15 individuals from China, respectively

Locus	Russia(*n* = 9)			China(*n* = 15)		
	*H*_E_	*H*_O_	PIC	*H*_E_	*H*_O_	PIC
FCA32	0.47	0.43	0.39	0.26	0.14	0.23
FCA69	0.70	0.56	0.59	0.44	0.40	0.39
FCA77	0.63	0.67	0.54	0.54	0.67	0.44
FCA105	0.65	0.56	0.54	0.60	0.73	0.52
FCA44	0.50	0.56	0.40	0.68	0.80	0.61
FCA161	0.76	0.78	0.67	0.74	0.80	0.66
FCA176	0.60	0.57	0.46	0.65	0.83	0.54
FCA220	0.57	0.78	0.47	0.60	0.80	0.49
Mean	0.61	0.61	0.51	0.57	0.65	0.49

Note

*H*_E_: expected heterozygosity; *H*_O_: observed heterozygosity; PIC: polymorphism information content.

In this study, individuals of B1–B15 were sampled from China and B16–B23 were sampled from Russia and more than 65% of assigned individuals match the geographic sampling site (Table 3). Bayesian clustering analysis by STRUCTURE revealed *K* = 2, using the K criterion method (Evanno et al., 2005). However, individual migration was not successfully detected. Individuals with a Q score falling between 0.2 and 0.8 can be considered as potentially admixed (Bergl & Vigilant, 2007), and individuals in this study with Q scores falling between 0.4 and 0.6 were taken as potentially admixed individuals. Structure cluster and geographical sampling locality were both used as prior population information to identify the migration of individuals. Unlike structure, GENECLASS does not assume that all source populations were sampled, so it is more effective at detecting immigrants from nonsampled populations (Bergl et al., 2007).

**Table 3:** Individual detection of Amur tiger origin and dispersal in the study area

Sample code	GO	Q		GLOP	GHAP	F_0_	SMP	FC
B1	China	0.564	0.436	Russia	0.831	0.995	0.09	MS
B2	China	0.436	0.564	China	0.981	0.452	0.041	–
B3	China	0.461	0.539	China/Russia	0.936/0.849	0.453	0.048	–
B4	China	0.488	0.512	China/Russia	0.541/0.485	0.451	0.047	–
B5	China	0.429	0.571	China/Russia	0.971/0.942	0.451	0.042	–
B6	China	0.423	0.577	China/Russia	0.922/0.993	0.962	0.053	MU/MS
B7	China	0.585	0.415	China	0.022	0.453	0.117	–
B8	China	0.435	0.565	China	0.992	0.453	0.04	–
B9	China	0.442	0.558	China/Russia	0.956/0.932	0.455	0.047	–
B10	China	0.558	0.442	China/Russia	0.455/0.417	0.453	0.052	–
B11	China	0.424	0.576	China	0.957	0.452	0.036	–
B12	China	0.435	0.565	China	0.990	0.432	0.038	–
B13	China	0.461	0.539	China	0.982	0.452	0.04	–
B14	China	0.467	0.533	China	0.964	0.452	0.044	–
B15	China	0.566	0.434	China	0.232	0.452	0.067	–
B16	Russia	0.431	0.569	Russia	0.772	0.924	0.055	–
B17	Russia	0.431	0.569	China/Russia	0.863/0.940	0.951	0.077	MS/MU
B18	Russia	0.438	0.562	Russia	0.996	0.361	0.055	–
B19	Russia	0.571	0.429	Russia	0.927	0.347	0.041	–
B20	Russia	0.577	0.423	Russia	0.928	0.341	0.041	–
B21	Russia	0.544	0.456	Russia	0.713	0.343	0.037	–
B22	Russia	0.576	0.424	Russia	0.405	0.379	0.041	–
B23	Russia	0.547	0.453	Russia	0.862	0.375	0.054	–

Notes

F_0_: GeneClass migrant probability; FC: final migrant/admixture classification; GHAP: GeneClass highest assignment probability; GLOP: GeneClass locality of highest probability assignment‐exclusion test; GO: geographic origin; MS: migrant source locality were determined; MU: migrant whose source locality could not be determined; Q: structure Q; SMP: structure migrant probability; “–” represents no results.

Three of the 23 sampled individuals (B1, B6, and B17) were identified as potential migrants with a probability that exceed 0.95 (F_0_) and a rate of dispersal of 13.04% (3/23). Two of them (male) sampled in China were suspected to be originally from Russia, while the other 1 (female) migrated in the opposite direction. The individual called B1 assigned strongly to a particular locality in the exclusion test, indicating a seemingly clear population of origin (Table 3). B6 may have originally come from Russia and settled in China with a permanent home range, since we collected its feces samples two consecutive years in the same region. Combined with the migrant detection results, B1 was assigned to Russia instead of its sample collection place in China. B6 and B17 were assigned equally to two localities, and after taking the detection results into consideration, we found that B6 may have been from Russia or another unknown origin and B17 was actually from China or another unknown origin. Six tiger individuals (B3, B4, B5, B6, B9, B10, and B17) had similar probabilities for several areas, as seen by the GeneClass analysis (GLOP). According to Bergl's standards, we did not detect any admixture individuals.

The geographical distance values from individuals to the boundary ranged from 0.37 to 219.267 km in China. In this research, the HL value was 0.299 ± 0.17 and the IR value ranged from −0.596 to 0.341. After screening the model, we finally used the Gamma and Lognormal distributions model, which showed that the relationship between geographical distance of the identified individuals to the boundary and HL or IR was not significant.

**Figure 3:**
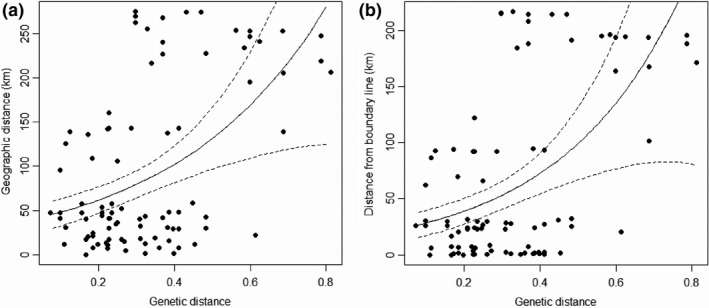
The relationships between geographic distance and genetic distance among individuals of Amur tiger in China (a), and distances from the Sino–Russia boundary line and genetic distances among individuals of Amur tiger in the eastern Changbai mountain in China (b)

The Nei's D_A_ distance values among different individuals ranged from 0.073 to 0.812 (0.332 ± 0.171), and the geographical distance values ranged from 77.99 to 274.908 (95.329 ± 92.832) km. The difference in the geographical distance from individual to the boundary ranged from 0.031 to 216.569 (68.391 ± 78.553 km). The correlation analysis of the Gamma and lognormal distributions model showed significant positive correlation between genetic distances and geographical distances, as well as the distances to the boundary (Figure 3).

## 4 DISCUSSION

It was reported that Amur tiger habitat had increased in China since the beginning of the Natural Forest Protection Project in 1998 (Jiang et al., 2017). In fact, Amur tigers had reappeared in many places in the interior lands of China in recent years (e.g., Huanan, Fangzheng, Linkou, Hailin, and Jiaohe forest areas, and part areas maintained by other forest bureaus), places where Amur tigers had disappeared for more than two decades. In this study, we found that population expansion was continuing westwards at a certain speed. It affirmed the progress made by the Chinese government in Amur tiger conservation, reflecting the Amur tiger habitat expansion in China. The study area includes in two main Amur tiger habitat patches in China, which were separated by cities, roads, and farmlands. Previous research found that an Amur tiger individual dispersed from the Laoyeling mountain patch, next to the border, to the interior Zhangguangcailing mountain patch. This finding was based on camera trap data. It was possible to grasp the connection between these two patches but much more work is required. Improving the habitat condition between these two patches and promoting the dispersal of the Laoyeling mountain population patch to disperse toward the Chinese interior was an effective measure and is vital for Amur tiger recovery and persistence in China.

The Amur tiger habitat in Changbai mountain in China was linked with southwest Primorye in Russia where 1 of the 2 Amur tiger populations lives in Russia. This population was limited in a small area by city and a railroad; thus, the successful dispersal from Russia to China was not only crucial to Amur tiger population recovery in China but also important for the local population to avoid inbreeding and over competition. The southwest Primorye population in Russia, as the source population for population recovery in the Changbai mountain and its genetic composition, is extremely important to the population in China (Henry et al., 2009; Miquelle et al., 2015). Understanding the population separation or gene exchange status across the international border is important for promoting the transboundary movement of Amur tigers and maintaining genetic biodiversity. Our study found that the difference in genetic diversity was slight (*p* > 0.05) and there was no distinct cluster between populations in China and Russia, as the all individuals identified had *Q* values between 0.4 and 0.5. There was evidently some individuals crossed the boundary based on molecular analysis. The macroapproach also found evidence of transboundary movement. The tracking of a young female tiger in Russia found that she crossed the national boundary at the Ussuri River and moved into China (Miquelle et al., 2006). Combining the outcomes of STRUCTURE and GENECLASS, we identified B1, B6, and B17 as potential migrants or migrant ancestry. The existence of potential migrants indicated the existence of potential individual exchange between Russia and China. However, only three potential individual migrants (or of migrant ancestry) in total reflected that there may still be obstacles hindering the transboundary movement of tigers. More international cooperation between China and Russia should be conducted in the Sino–Russian border areas to eliminate obstacles hindering the movement of the Amur tiger, and international ecological corridors should be constructed between China and Russia. Then, the transboundary movement of Amur tiger between China and Russia should be promoted.

Genetic distance and geographical distance of Amur tigers in China were significantly positively correlated, meaning that with the increase in geographical distance between individuals, kinship becomes more estranged. Geographical distance could influence population structure. The shortest distance from the individual to the boundary ranged from 0.37 to 219 km. Correlation analysis found no significant relationship between individual heterozygosity calculated by two methods and the distance to the boundary, since only long‐distance dispersal makes the most sense for changing genetic relationships (Trizio et al., 2005).

Our results comprehensively demonstrated that the migration of the Amur tiger from Russia to China and the dispersal further into China was possible, and it was occurring. But successful migration and dispersal were occasional and faced great challenges. There were two reasons that may contribute to this situation. First, previous research identified that cattle grazing was in competition with ungulates for food and space resources, which ultimately leads to a sharp reduction in prey for the Amur tiger. Second, the distribution of villages, roads, farmland, and other infrastructure has a negative effect on prey and on the Amur tiger itself. These factors also affect tiger movement and their ability to breed successfully in the environment (Li et al., 2017; Wang, Feng, et al., ). Besides continuing habitat conservation and transnational cooperation to promote the transboundary movement and dispersal of the Amur tiger, human disturbance should be strictly controlled. Moreover, female tigers are crucial for population recovery, and they need more resources for reproduction, so the dispersal and relocation of female individuals act as an indication of successful population dispersal because female Amur tiger cubs tend toward philopatry and usually set up a home range near their birthplace (Goodrich et al., 2010). Therefore, managers should pay careful attention while monitoring the population and distribution conditions of the Amur tiger, keep tracking the transboundary movement and dispersal dynamics, focusing especially on female individuals.

## 5 CONCLUSIONS

The Amur tiger population in China is in a stage of continuous growth. Individuals need to be provided with indispensable opportunities, and conservation managers need enough data concerning habitat and population dynamic assessments. This study combined the explicit spatial genetic analysis to explore Amur tiger dispersal both across the international boundary and into China. This study clarified the dispersal process of the Amur tiger into China and how genetic conditions benefit from dispersal. The potential trans‐boundary movement of Amur tiger was also assessed.

The study demonstrated that international cooperation and the continuous monitoring of spatial distribution and genetic information are critical to understand the dispersal limitations and recovery effectiveness of Amur tigers in both countries. Based on these results, we believe that the free movements of Amur tigers from border areas further into China, as well as between China and Russia, were limited by some unknown disturbance factors. Thus, international cooperation between China and Russia should be strengthened, and the habitat restoration in China should continue. Furthermore, ecological corridors between China and Russia and habitat patches in the Laoyeling mountain and the Zhangguangcailing mountain should be constructed to create viable migration opportunities for the Amur tiger, in order to promote individual exchange within inner China, and keep sustainable tiger population in the Sino–Russian border areas. Meanwhile, to keep track of the population and dispersal dynamics of the Amur tiger by different methods were important references to assist with timely adjustments to conservation strategies. This study provided a good example of joint research for the continual monitoring of the recovery of other species in both China and Russia.

## CONFLICT OF INTEREST

None declared.

## AUTHORS’ CONTRIBUTION

G. J., A. K., and J. M. conceived and designed this study. Y. N. and G. J. wrote the manuscript and carried out field and laboratory work with C. M., V. Y. G., J. Q., and M. W.

## Data Availability

Source code files of the models: Dryad doi: https:\\doi.org\10.5061/dryad.p8c7dg7. The data will be deposited to Dryad if the paper is accepted.
