# Supplementary material for: Dispersal of Amur tiger from spatial distribution and genetics within the eastern Changbai mountain of China
Source: Ecol Evol. 2019 Feb 18;9(5):2415–24. doi: 10.1002/ece3.4832 (PMC6405893; doi:10.1002/ece3.4832)
Supplement: Supplementary file 1 [file ECE3-9-2415-s001.docx]

**Table S1**

| No. | FCA008 | | FCA032 | | FCA069 | | FCA077 | | FCA105 | | FCA005 | | FCA043 | | FCA290 | | FCA090 | | FCA091 | | FCA094 | |
| --- | --- | --- | --- | --- | --- | --- | --- | --- | --- | --- | --- | --- | --- | --- | --- | --- | --- | --- | --- | --- | --- | --- |
| 1 | 133 | 133 | 0 | 0 | 97 | 99 | 0 | 0 | 209 | 209 | 150 | 154 | 126 | 126 | 0 | 0 | 105 | 109 | 0 | 0 | 0 | 0 |
| 2 | 135 | 135 | 186 | 190 | 99 | 101 | 148 | 162 | 197 | 205 | 154 | 158 | 126 | 126 | 213 | 213 | 107 | 107 | 207 | 209 | 207 | 209 |
| 3 | 135 | 135 | 190 | 190 | 99 | 101 | 148 | 148 | 197 | 197 | 154 | 156 | 126 | 126 | 205 | 213 | 107 | 107 | 0 | 0 | 209 | 209 |
| 4 | 135 | 135 | 190 | 190 | 99 | 99 | 146 | 146 | 197 | 197 | 158 | 162 | 130 | 130 | 0 | 0 | 111 | 111 | 161 | 161 | 0 | 0 |
| 5 | 133 | 133 | 0 | 0 | 105 | 105 | 0 | 0 | 195 | 195 | 152 | 154 | 124 | 124 | 0 | 0 | 105 | 105 | 0 | 0 | 0 | 0 |
| 6 | 137 | 137 | 0 | 0 | 0 | 0 | 150 | 150 | 199 | 199 | 156 | 158 | 128 | 128 | 0 | 0 | 109 | 109 | 0 | 0 | 211 | 211 |
| 7 | 135 | 135 | 190 | 190 | 99 | 99 | 148 | 162 | 205 | 209 | 156 | 158 | 120 | 130 | 221 | 221 | 109 | 109 | 0 | 0 | 211 | 211 |
| 8 | 135 | 135 | 188 | 190 | 99 | 99 | 162 | 162 | 209 | 209 | 156 | 158 | 124 | 130 | 213 | 213 | 109 | 109 | 0 | 0 | 209 | 209 |
| 9 | 135 | 135 | 190 | 190 | 99 | 99 | 148 | 162 | 205 | 209 | 154 | 156 | 126 | 130 | 219 | 221 | 109 | 109 | 0 | 0 | 209 | 211 |
| 12 | 135 | 135 | 190 | 192 | 95 | 99 | 148 | 148 | 209 | 209 | 154 | 156 | 126 | 130 | 211 | 211 | 109 | 109 | 0 | 0 | 0 | 0 |
| 13 | 135 | 135 | 190 | 190 | 95 | 99 | 148 | 162 | 209 | 209 | 154 | 158 | 120 | 126 | 217 | 217 | 107 | 109 | 0 | 0 | 211 | 211 |
| 14 | 135 | 135 | 190 | 190 | 99 | 99 | 148 | 162 | 207 | 209 | 152 | 156 | 126 | 126 | 0 | 0 | 107 | 111 | 0 | 0 | 0 | 0 |
| 15 | 135 | 135 | 190 | 190 | 99 | 99 | 148 | 162 | 197 | 209 | 154 | 158 | 120 | 130 | 205 | 213 | 109 | 109 | 0 | 0 | 209 | 209 |
| 16 | 135 | 135 | 190 | 190 | 101 | 101 | 148 | 162 | 207 | 209 | 154 | 156 | 126 | 130 | 0 | 0 | 107 | 109 | 0 | 0 | 211 | 211 |
| 17 | 135 | 135 | 188 | 190 | 95 | 99 | 148 | 148 | 209 | 209 | 156 | 156 | 130 | 130 | 213 | 213 | 107 | 109 | 0 | 0 | 209 | 209 |
| 18 | 135 | 135 | 0 | 0 | 99 | 99 | 148 | 148 | 0 | 0 | 158 | 158 | 0 | 0 | 0 | 0 | 109 | 109 | 0 | 0 | 0 | 0 |
| 19 | 135 | 135 | 188 | 190 | 99 | 99 | 148 | 162 | 205 | 209 | 156 | 158 | 120 | 130 | 213 | 221 | 109 | 109 | 165 | 165 | 209 | 211 |

Note: number in red mean that a homozygote with less than 3 times and less than2times for a heterozygote; No.19 was the positive control which isolated DNA from tiger blood.

**Continue Table S1**

| No. | FCA044 | | FCA126 | | FCA161 | | FCA176 | | FCA220 | | FCA441 | | FCA293 | | FCA304 | | FCA310 | | FCA391 | | FCA211 | |
| --- | --- | --- | --- | --- | --- | --- | --- | --- | --- | --- | --- | --- | --- | --- | --- | --- | --- | --- | --- | --- | --- | --- |
| 1 | 115 | 119 | 0 | 0 | 181 | 181 | 0 | 0 | 201 | 201 | 148 | 156 | 0 | 0 | 0 | 0 | 0 | 0 | 0 | 0 | 0 | 0 |
| 2 | 115 | 115 | 148 | 148 | 185 | 185 | 217 | 217 | 201 | 201 | 148 | 148 | 198 | 206 | 130 | 138 | 129 | 129 | 215 | 215 | 167 | 167 |
| 3 | 115 | 123 | 148 | 148 | 179 | 187 | 215 | 217 | 195 | 201 | 140 | 156 | 206 | 206 | 136 | 138 | 129 | 129 | 215 | 215 | 167 | 167 |
| 4 | 115 | 119 | 146 | 148 | 185 | 187 | 217 | 217 | 201 | 201 | 148 | 148 | 198 | 206 | 0 | 0 | 129 | 131 | 215 | 215 | 0 | 0 |
| 5 | 117 | 117 | 152 | 152 | 189 | 189 | 221 | 221 | 205 | 205 | 152 | 152 | 204 | 204 | 0 | 0 | 0 | 0 | 0 | 0 | 0 | 0 |
| 6 | 115 | 123 | 148 | 148 | 179 | 187 | 215 | 217 | 195 | 201 | 140 | 156 | 204 | 204 | 0 | 0 | 0 | 0 | 211 | 211 | 0 | 0 |
| 7 | 119 | 123 | 148 | 148 | 181 | 187 | 217 | 219 | 195 | 201 | 156 | 156 | 206 | 206 | 136 | 138 | 129 | 131 | 215 | 215 | 165 | 167 |
| 8 | 117 | 119 | 148 | 148 | 181 | 187 | 217 | 217 | 195 | 201 | 148 | 156 | 206 | 206 | 136 | 138 | 131 | 131 | 215 | 215 | 165 | 167 |
| 9 | 119 | 123 | 148 | 148 | 181 | 185 | 215 | 217 | 195 | 201 | 148 | 156 | 206 | 206 | 138 | 138 | 129 | 129 | 215 | 215 | 167 | 169 |
| 12 | 119 | 119 | 148 | 148 | 183 | 189 | 217 | 219 | 197 | 203 | 152 | 160 | 208 | 208 | 0 | 0 | 0 | 0 | 0 | 0 | 167 | 169 |
| 13 | 119 | 119 | 148 | 148 | 181 | 181 | 215 | 217 | 201 | 201 | 148 | 156 | 198 | 206 | 136 | 138 | 129 | 131 | 215 | 215 | 165 | 165 |
| 14 | 119 | 119 | 146 | 148 | 181 | 181 | 217 | 217 | 199 | 199 | 0 | 0 | 206 | 206 | 0 | 0 | 129 | 131 | 0 | 0 | 0 | 0 |
| 15 | 117 | 119 | 148 | 148 | 179 | 187 | 215 | 217 | 195 | 201 | 148 | 156 | 206 | 206 | 130 | 136 | 131 | 131 | 215 | 215 | 167 | 167 |
| 16 | 115 | 119 | 148 | 148 | 179 | 185 | 217 | 217 | 197 | 197 | 180 | 180 | 198 | 206 | 130 | 130 | 129 | 131 | 215 | 219 | 0 | 0 |
| 17 | 119 | 119 | 148 | 148 | 181 | 187 | 0 | 0 | 195 | 201 | 148 | 148 | 206 | 206 | 136 | 136 | 129 | 129 | 215 | 215 | 0 | 0 |
| 18 | 0 | 0 | 148 | 148 | 183 | 183 | 217 | 219 | 197 | 203 | 152 | 156 | 208 | 208 | 0 | 0 | 131 | 131 | 0 | 0 | 167 | 167 |
| 19 | 119 | 123 | 146 | 148 | 181 | 187 | 215 | 217 | 195 | 201 | 148 | 156 | 206 | 206 | 136 | 138 | 129 | 131 | 215 | 215 | 165 | 167 |

Note: number in red mean that a homozygote with less than 3 times and less than2times for a heterozygote; No.19 was the positive control which isolated DNA from tiger blood.
